# Monthly Record of Deaths in the City of Buffalo

**Published:** 1854-09

**Authors:** Jas. Newman

**Affiliations:** Health Physician


ART. VI.—*Record of Mortality in the City of Buffalo, for the Month of July*, 1854. By J. M. NEWMAN, Health Physician.

| DISEASES. | No. | Males. | Females. |
|---|---|---|---|
| Abscessus Genu, | 1 | 1 | |
| Accidens, | 6 | 4 | 1 |
| Apoplexia, | 1 | | 1 |
| Asphyxia Immersorum, | 11 | 7 | 1 |
| Bronchitis, | 1 | 1 | |
| Cancer Mammæ, | 1 | | 1 |
| Cholera Asphyxia, | 159 | 89 | 59 |
| " Infantum, | 44 | 20 | 22 |
| " Morbus, | 7 | 2 | 4 |
| Coup De Soleil, | 1 | 1 | |
| Cyanosis, | 1 | | 1 |
| Cynanche Trachealis, | 1 | | 1 |
| Cystitis, | 1 | 1 | |
| Debilitas, | 15 | 6 | 8 |
| Diarrhœa, | 27 | 11 | 13 |
| Dysenteria, | 10 | 4 | 6 |
| Eclampsia, | 26 | 10 | 11 |
| Enteritis, | 1 | 1 | |
| Febris Biliosa, | 2 | 1 | 1 |
| " Puerperal, | 1 | | 1 |
| " Scarlatina, | 4 | 1 | 3 |
| " Typhus, | 5 | 3 | 1 |
| Hydrops, | 1 | | |
| Hydrops Cerebri, | 9 | 4 | 5 |
| Hepatitis, | 1 | 1 | |
| Hyperæmia Cerebri, | 5 | 4 | 1 |
| " Pulmonalis, | 2 | 1 | 1 |
| Intemperantia et Delir. Tremens, | 4 | 3 | |
| Marasmus, | 3 | 1 | |
| Metritis, | 1 | | 1 |
| Morbus Cordis, | 1 | | 1 |
| Natus Mortuus, | 11 | | |
| Negligentia, | 2 | 1 | |
| Parturitio, | 1 | | 1 |
| Pertussis, | 1 | 1 | |
| Peritonitis, | 1 | | 1 |
| Phthisis Pulmonalis, | 22 | 10 | 11 |
| Phrenitis, | 1 | | 1 |
| Phlegmons, | 1 | | 1 |
| Pneumonia, | 6 | 5 | 1 |
| Rubeola, | 2 | 1 | 1 |
| Scrophula, | 2 | 2 | |
| Senectus, | 2 | 2 | |
| Suicidium, | 1 | 1 | |
| Suspectus Homicidium, | 1 | | 1 |
| Spina Bifida, | 1 | | 1 |
| Tetanus, | 1 | 1 | |
| Ignotus, | 20 | 11 | 8 |
| | 429 | | |

| | |
|---|---|
| Males, | 212 |
| Females, | 171 |
| Sex not given, | 46 |
| Total, | 429 |

REGISTER OF MORTALITY—(Continued.)

AGES.

| | | | |
|---|---|---|---|
| Still-born, | 11 | Between 6 years and 12 years, | 22 |
| 1 day, | 2 | " 12 " " 20 " | 16 |
| Between 1 day and 15 days, | 14 | " 20 " " 30 " | 54 |
| " 15 days and 30 days, | 7 | " 30 " " 40 " | 34 |
| " 1 month and 3 months, | 24 | " 40 " " 50 " | 40 |
| " 3 months and 6 months, | 22 | " 50 " " 60 " | 25 |
| " 6 " " 9 " | 21 | " 60 " " 70 " | 9 |
| " 9 " " 12 " | 26 | " 70 " " 80 " | 4 |
| " 1 year and 3 years, | 47 | " 80 " " 90 " | 2 |
| " 3 years and 6 years, | 37 | Ages not given, | 12 |
| Total, | | | 429 |

NATIVITY.

| | | | |
|---|---|---|---|
| American, | 103 | Canadian, | 1 |
| English, | 10 | Italian, | 1 |
| Scotch, | 1 | Swiss, | 4 |
| Irish, | 48 | Norwegian, | 5 |
| German, | 174 | Colored, | 4 |
| French, | 6 | Country not given, | 72 |
| Total, | | | 429 |

We are still unable to make our tables as perfect as we could wish, owing to the imperfect and careless manner of filling up the certificates upon which the permits are issued. A little more attention to the subject might remedy these imperfections. It is readily conceded that in reference to the age and nativity of the person dying it may not at all times be possible to obtain accurate information, but one would suppose that the question of sex might always be answered. Many of the certificates are now so written as to render it impossible to ascertain this fact. This arises from certifying in children that it is "*the child of Mr. or Mrs.* ——;" and in adults giving an *initinl letter* of the christian name only, without any prefix or suffix which will afford any enlightenment upon the subject. In the physician this carelessness is almost unpardonable.

